# *In-Vivo* Fusion of Human Cancer and Hamster Stromal Cells Permanently Transduces and Transcribes Human DNA

**DOI:** 10.1371/journal.pone.0107927

**Published:** 2014-09-26

**Authors:** David M. Goldenberg, Robert J. Rooney, Meiyu Loo, Donglin Liu, Chien-Hsing Chang

**Affiliations:** 1 Garden State Cancer Center, Center for Molecular Medicine and Immunology, Morris Plains, New Jersey, United States of America; 2 Immunomedics, Inc., Morris Plains, New Jersey, United States of America; 3 Genome Explorations, Inc., Memphis, Tennessee, United States of America; Witten/Herdecke University, Germany

## Abstract

After demonstrating, with karyotyping, polymerase chain reaction (PCR) and fluorescence *in-situ* hybridization, the retention of certain human chromosomes and genes following the spontaneous fusion of human tumor and hamster cells *in-vivo*, it was postulated that cell fusion causes the horizontal transmission of malignancy and donor genes. Here, we analyzed gene expression profiles of 3 different hybrid tumors first generated in the hamster cheek pouch after human tumor grafting, and then propagated in hamsters and in cell cultures for years: two Hodgkin lymphomas (GW-532, GW-584) and a glioblastoma multiforme (GB-749). Based on the criteria of MAS 5.0 detection *P*-values ≤0.065 and at least a 2-fold greater signal expression value than a hamster melanoma control, we identified 3,759 probe sets (ranging from 1,040 to 1,303 in each transplant) from formalin-fixed, paraffin-embedded sections of the 3 hybrid tumors, which unambiguously mapped to 3,107 unique Entrez Gene IDs, representative of all human chromosomes; however, by karyology, one of the hybrid tumors (GB-749) had a total of 15 human chromosomes in its cells. Among the genes mapped, 39 probe sets, representing 33 unique Entrez Gene IDs, complied with the detection criteria in all hybrid tumor samples. Five of these 33 genes encode transcription factors that are known to regulate cell growth and differentiation; five encode cell adhesion- and transmigration-associated proteins that participate in oncogenesis and/or metastasis and invasion; and additional genes encode proteins involved in signaling pathways, regulation of apoptosis, DNA repair, and multidrug resistance. These findings were corroborated by PCR and reverse transcription PCR, showing the presence of human alphoid (α)-satellite DNA and the *F11R* transcripts in additional tumor transplant generations. We posit that *in-vivo* fusion discloses genes implicated in tumor progression, and gene families coding for the organoid phenotype. Thus, cancer cells can transduce adjacent stromal cells, with the resulting progeny having permanently transcribed genes with malignant and other gene functions of the donor DNA. Using heterospecific *in-vivo* cell fusion, genes encoding oncogenic and organogenic traits may be identified.

## Introduction

Primary human tumor transplants, particularly to immunosuppressed rodents, such as nude and NOD/SCID mice, are used as preclinical models for evaluating tumor biology and drug sensitivity [1]–[7]. These studies are based on the supposition that such xenografts retain the properties and critical genotypes of their donor tumors, thus being predictive for clinical translation. However, we and others have demonstrated that such transplants can induce tumors in their rodent recipients, such as golden hamsters [8]–[10], nude/SCID mice [11]–[24], and immunosuppressed rats [25], although infrequently (either because of low incidence or rare testing). One plausible explanation is the horizontal transfer of oncogenic DNA [25]–[27]. Indeed, lateral oncogenesis between tumor and its stromal cells can be traced back to Ehrlich and Apolant in 1905, who showed that stromal cells of a tumor can become a sarcoma when a carcinoma is grafted in mice, and in fact the authors conjectured that a chemical factor was implicated [28]. Seventy-six years later, a human carcinoma transplanted to nude mice also was reported to induce fibrosarcomas that killed the nude mouse recipients and could propagate as malignant tumors in immune competent mice of the same genetic background [12]. In addition, a human ovarian cancer transplant to nude mice showed two cancer populations, an epithelial and a sarcomatous, the former showing human and the latter murine properties [14], thus suggesting lateral transduction or DNA transfer. Only the murine sarcoma cells, which were postulated to be induced by the human carcinoma cells, were metastatic and lethal in nude mice or immunocompetent mice of the same genetic background [14]. This induction of stromal tumors in host animals after xenotransplantation of human epithelial cancers has been confirmed by others [15]–[25], thus suggesting that cancer xenografts be carefully evaluated for horizontal oncogenesis [13], [24]. How this transformation or induction occurred was not elucidated, but a viral role has been discussed [17].

In some of these experiments involving primary human tumor transplants, transfer of functional human genetic information by *in-vivo* cell hybridization of the donor tumor and recipient host cells, showing chromosomal, immunological, or genetic features of both partners [9], [29]–[33], was proposed as the mechanism for induction of these tumors that exhibited highly invasive and metastatic behavior in their animal hosts [34], [35]. For example, we reported that after long-term propagation of human-hamster hybrid tumors derived from a glioblastoma multiforme [33] and two Hodgkin lymphomas, human DNA and genes could be confirmed by fluorescence *in-situ* hybridization (FISH) and polymerase chain reaction (PCR), and their donor organoid features by histology [36], [37]. Translation of some of these gene products was found by immunohistochemistry (IHC) in the glioblastoma multiforme transplants, even after propagation for over a year [36].

These results indicate that human genes can remain functional within human-hamster hybrid tumors propagated in the animal host, emphasizing the horizontal transmission of human DNA implicated with malignancy and the organoid features of the original patient donor tumors. However, the scope of human DNA transduced and transcribed in these interspecies hybrid cells has not been investigated. Accordingly, we examined (*i*) if such formalin-fixed, paraffin-embedded (FFPE) tumor grafts, which were stored for over 40 years since they were made, could be tested globally for the expression of transcribed human genes, (*ii*) if human genes are retained during long-term serial passage, and (*iii*) if there are specific human gene families indigenous to these human-hamster hybrid tumors. By using tumors and hosts of different species, we are able to identify each party's genetic contribution, which is especially problematic when attempting to prove cell-cell fusion in humans, whether involving normal-normal, malignant-normal, or malignant-malignant fusions.

We postulate that these results of heterospecific fusions provide a general mechanism of tumor DNA transfer to stromal cells that results in genetic instability, heterogeneity, and aneuploidy, leading to stable genomic changes associated with cancer progression, while also retaining the tumor's original organoid phenotype, as well as other genes derived from the donor human tumor. This merging of tumor and normal genomes into a new population of malignant hybrid cells could be a mechanism whereby a cancer escapes host immunity by reducing the immunological disparity between the tumor and its host [34], [35]. Various aspects of the role of cell-cell fusion in cancer are now gaining increased attention [35], [38]–[47].

## Results

**Figure 1:**
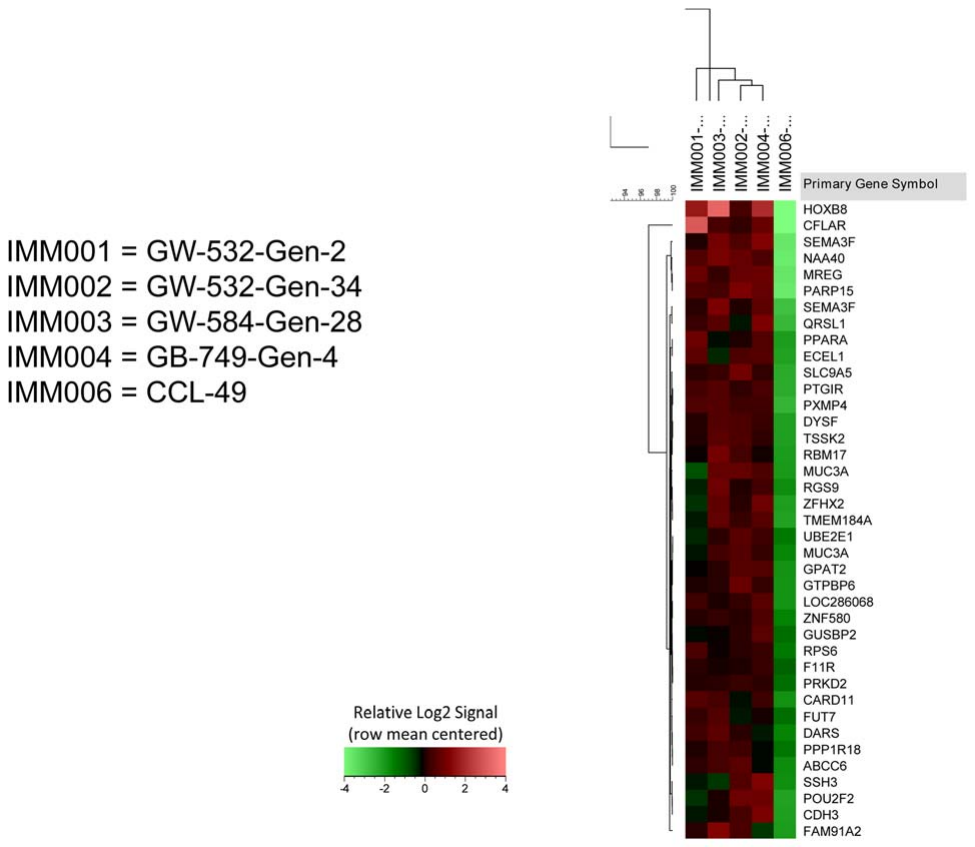
Clustered heat map of the 39 human probe sets detected in all four hybrid tumor samples. The heat map depicts expression signal values for 39 Affymetrix Human U133_X3P probe sets detected in FFPE sections from all four hybrids tested (IMM001-004) and a hamster control (IMM006). Prior to unsupervised hierarchical clustering, the MAS 5.0 signal values were log2-transformed and row mean centered. Samples were clustered by Complete Linkage based on Pearson correlation; probe sets were clustered by Complete Linkage based on Euclidean distance. Criteria for detectable human gene expression included MAS 5.0 Detection p-values ≤0.065 in the hybrid sample and >0.065 in the hamster control, and ≥2-fold increased signal in the hybrid sample vs. the hamster control.

Human mRNA transcripts present in each of four different human-hamster hybrid tumor FFPE samples (Table 1) were identified by analysis of total RNA, in comparison to a control hamster melanoma line (CCL-49), using Affymetrix Human U133 X3P arrays. Probe sets with MAS 5.0 detection *P*-values ≤0.065 in a hybrid sample, a detection *P*-value>0.065 in the hamster control, and an expression signal value that was at least 2-fold greater in the hybrid sample than in the hamster control, were considered to represent expressed human gene transcripts. Using these criteria, we identified a total of 3759 probe sets (ranging from 1040 to 1303 probe sets in at least one hybrid sample), which unambiguously mapped to 3107 unique Entrez Gene IDs (Table S1), representing genes from all human chromosomes. Among these, 39 probe sets passed all of the expression criteria in all four hybrid specimens (Figure 1, Table 2), with 34 probe sets detecting 33 unique Entrez Gene IDs (Table S2), two probe sets detecting either *MUC3A* or *MUC1B*, and the remaining probe sets detecting an uncharacterized gene (*LOC286068*), *GUSBP2* or mutlple *GUSB* pseudogenes, and *FAM91A2* or multiple uncharacterized genes. Thus, at least 33 unique human genes were transcribed in these FFPE tissues from 3 different human tumor xenografts representing different transplant generations, including two for GW-532, propagated serially for months to years as highly metastatic tumors.

**Table 1:** Characteristics of test articles used in the microarray study.

RNA sample^a^	Transplant	Generation	Primary tumor
IMM001	GW-532	Gen-2^b^	Hodgkin lymphoma
IMM002	GW-532	Gen-34	Hodgkin lymphoma
IMM003	GW-584	Gen-28^c^	Hodgkin lymphoma
IMM004	GB-749	Gen-4^d^	Glioma
IMM006	NA^e^	NA^e^	Hamster melanoma

a Prepared from FFPE specimens as indicated, except IMM006, which was prepared from CCL-49, a Syrian golden hamster melanoma cell line acquired from ATCC.

b Human genes of *CD74*, *CXCR4*, *CD19*, *CD79b*, and *VIM* were detected by PCR (Ref. 37).

c Human genes of *CD74*, *CXCR4*, *CD20*, and *CD79b* were detected by PCR (Ref. 37).

d The expression of CD74, CXCR4 and PLAGL2 were detected by IHC staining (Ref. 36).

e Not applicable.

**Table 2:** The 39 probe sets determined to be positive in all hybrid FFPE specimens.

Probe Set ID	Primary Gene Symbol	Chromosomal Location
Hs.183274.0.A1_3p_at	*HOXB8*	17q21.3
g2429159_3p_a_at	*CFLAR*	2q33-q34
Hs2.120250.2.S1_3p_a_at	*PARP15*	3q21.1
35666_3p_at	*SEMA3F*	3p21.3
g13376118_3p_at	*NAA40*	11q13.1
Hs.79741.1.S1_3p_at	*MREG*	2q35
4871689C_3p_s_at	*SEMA3F*	3p21.3
Hs.210778.1.A1_3p_at	*QRSL1*	6q21
Hs2.132171.1.S1_3p_x_at	*SLC9A5*	16q22.1
g4502722_3p_at	*CDH3*	16q22.1
g5454081_3p_at	*RBM17*	10p15.1
Hs.241205.0.S1_3p_a_at	*PXMP4*	20q11.22
Hs.147381.0.A1_3p_at	*POU2F2*	19q13.2
g8923482_3p_s_at	*SSH3*	11q13.2
Hs.128691.0.S1_3p_at	*ZFHX2*	14q11.2
g12652612_3p_at	*PPARA*	22q13.31
Hs.126067.0.A1_3p_at	*TMEM184A*	7p22.3
1555620_3p_a_at	*PTGIR*	19q13.3
g4758231_3p_x_at	*ECEL1*	2q37.1
Hs.103978.0.S1_3p_x_at	*TSSK2*	22q11.21
g6912587_3p_at	*GTPBP6*	Xp22.33; Yp11.32
g4506520_3p_a_at	*RGS9*	17q24
g4503430_3p_at	*DYSF*	2p13.3
Hs.146084.0.A1_3p_at	*GPAT2*	2q11.1
g12382772_3p_at	*CARD11*	7p22
Hs.274260.2.S1_3p_at	*ABCC6*	16p13.1
g12653688_3p_a_at	*DARS*	2q21.3
g7705880_3p_a_at	*ZNF580*	19q13.42
Hs.163546.0.A1_3p_x_at	*UBE2E1*	3p24.2
g12751054_3p_s_at	*RPS6*	9p21
Hs.325905.0.A1_3p_x_at	*FUT7*	9q34.3
Hs.101150.0.A1_3p_at	*PPP1R18*	6p21.3
241669_3p_x_at	*PRKD2*	19q13.3
g11065890_3p_a_at	*F11R*	1q21.2-q21.3
1568609_3p_s_at	*FAM91A2*	1q21.1
Hs.129782.1.S1_3p_a_at	*MUC3A*	7q22
Hs2.376165.1.S1_3p_at	*LOC286068*	8q11.21
Hs.129782.0.S1_3p_a_at	*MUC3A*	7q22
g5803174_3p_x_at	*GUSBP2*	5q13///13.2///6p21

As listed in Table S3, transcripts of the genes expressed in all four hybrid samples include five encoding transcription factors that are known to regulate cell growth and differentiation (*HOXB8, PPARA, POU2F2, ZFHX2, and ZNF580*), and five encoding cell adhesion and transmigration-associated proteins that participate in tumorigenesis and/or invasion/metastasis (*CDH3, FUT7, F11R, MUC3A, and SEMA3F*). In addition, genes whose products are associated with signaling pathways, regulation of apoptosis, DNA repair, and multidrug resistance, also were identified (namely, *PRKD2, ECEL1*, *CARD11, CFLAR, PARP15*, and *MRP6*).

Recognizing that the degraded nature of the FFPE RNA and the high background of hamster RNA in the FFPE hybrid samples could interfere with the sensitivity of MAS 5.0 detection *P*-values, we relaxed the detection *P*-value criterion by requiring a detection *P*-value ≤0.065 in only one of the four hybrid samples, instead of all four, and produced a larger list of human genes that potentially were commonly expressed in all of the hybrid samples. This second list contained 1120 probe sets, representing 982 unique Entrez Gene IDs (Table S4). These results indicate the presence of genes for CD20 (*MS4A1*), CD22, and CD44 (signaling component of the macrophage migration inhibitor factor (MIF)-CD74-CD44 receptor complex), thus corroborating the previous PCR results for the presence of CD20 and, also, CD74 genes in the GW-532 and GW-584 lymphoma hybrid tumors [36], [37]. A number of other human genes, such as those encoding CD24, CD27, CD47, CD52, CD84, CD151, and tenascin XB (*TNXB*), were found to be transcribed in these hybrid cell lines when the detection *P*-value criterion was relaxed (Table S4).

Pathway enrichment analysis of the larger, relaxed, common gene set and the individual gene sets from each of the four hybrid samples was performed with Webgestalt [48], [49], using the KEGG [50]–[52], and Pathway Commons databases [53], to identify similar pathways that are commonly represented in all four samples of the three hybrid tumors (Table S5). Pathways that were enriched in all five gene sets (the large common gene set and the four individual hybrid sample gene sets) fall into two general categories related to cell-cell communication/focal adhesion/cell junctions/ECM (extracellular matrix) interactions, and cytokine or growth factor signal transduction (including various ErbB signaling pathways). Pathways in two other general categories related to nuclear hormone receptors and MHC antigen processing/presentation were enriched in four of the five gene sets. Enrichment analysis using the DAVID Bioinformatics database [54], [55] identified six functional annotation clusters that were represented in all five gene sets: embryonic morphogenesis, cyclic AMP/adenylate cyclase activity, mitosis/ubiquitin-mediated proteolysis, nuclear hormone receptors, lymphocyte proliferation/activation, and apoptosis (Table S6). These results, from both the pathway and functional enrichment analyses, indicate that the various sets of human genes expressed in each hybrid tumor sample affect related cellular processes, and thereby likely produce similar effects on cellular function and growth.

**Figure 2:**
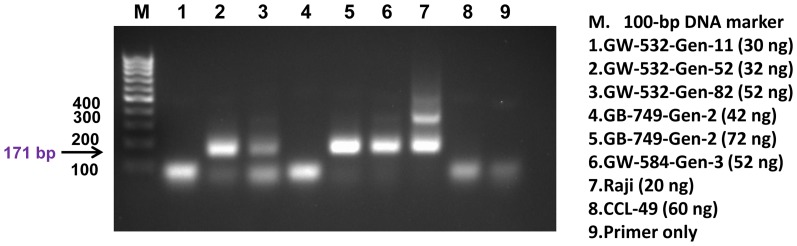
PCR of human alpha satellite DNA. The presence of human DNA was demonstrated by the detection of the 171-bp product in GW-532 generation 52 (lane 2), GW-532 generation 82 (lane 3), GB-749 generation 2 (lane 5), and GW-584 generation 3 (lane 6), but not in the negative control of hamster melanoma, CCL-49 (lane 8). The 171-bp and its higher oligomers were detected in the positive control of human Raji lymphoma cells (lane 7). The experimental conditions and the nominal amount of DNA used for each sample are indicated.

**Figure 3:**
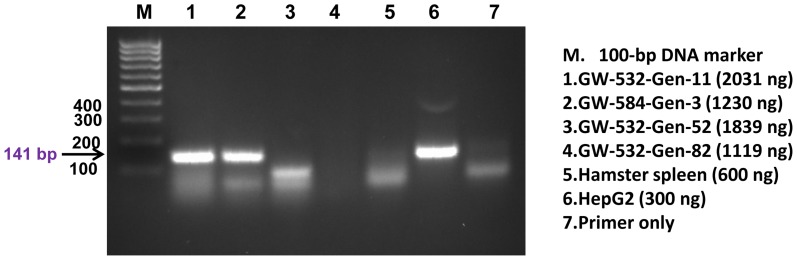
One-step reverse transcription PCR. The mRNA transcripts of *the F11R* gene were detectable in GW-532 generation 11 (lane 1), GW-584 generation 3 (lane 2), and the positive control of human HepG2 cells (lane 6), but not in the negative control hamster spleen cells (lane 5). The experimental conditions and the nominal amount of RNA used for each sample are indicated.

To further corroborate the microarray findings, PCR was performed on six additional FFPE tissue samples: three from GW-532 (generations 11, 52, and 82), one from GW-584 (generation 3), and two from GB-749 (both of generation 2), to assess the presence of human DNA in these tissue blocks, using a pair of primers directed to the 171-bp monomer of human alpha satellite DNA [56]. As shown in Figure 2, four of the 6 samples (GW-532 generations 52 and 82, GW-584 generation 3, and GB-749 generation 2) were positive for the expected PCR product of human αlpha satellite DNA (the 171-bp), which was detected also in the DNA of human lymphoma Raji cells (positive control), but not in the DNA of CCL-49 hamster melanoma cells (negative control). Moreover, we were able to confirm the expression of the *F11R* gene detected by the cDNA microarray studies in two of the six samples by one-step reverse transcription-PCR, using human hepatic cancer HepG2 cells as the positive control [57]. As shown in Figure 3, the presence of a 141-bp band was prominent in both GW-532 generation 11 and GW-584 generation 3, as well as in human HepG2 cells (positive control), but not in the tissue of a hamster spleen (negative control). These results were confirmed in a repeat experiment (Figure S1), using CCL-49 cells as the negative control.

## Discussion

In this study, we utilized human gene expression microarrays to provide further evidence that human genes can remain functional within metastatic human-hamster hybrid tumors propagated in the animal host, and corroborated such findings with additional samples showing the presence of human alphoid (α) satellite DNA and the *F11R* transcripts by PCR and reverse transcription-PCR, respectively. Our results demonstrate that human tumors transplanted to rodents can merge their DNA with the genome of the animal host, as an example of the larger program of tumor-stromal crosstalk. Cancer cells depend and are influenced by their “soil” or stromal microenvironment [58]–[60], but it is also known that there can be genetic interchange [61], [62]. The reciprocal horizontal transfer of genetic material between stromal and tumor cells could explain the heterogeneity and genetic diversity and evolution of cancer cell populations, not only between different patient tumors of the same cancer type, but even different tumors of the same patient, as observed in genetic analyses of human tumor specimens [63]–[65]. Cell-cell fusion enables the transfer of chromosomes and genetic material from one cell to another, and has been shown to result in viable hybrid progeny capable of replication for different periods, but usually not long-term or as permanent cell lines [66]. By using heterospecific cell-cell fusion *in-vivo*, genes controlling oncogenesis and organoid traits in the donor cancer cells may be elucidated in the fused progeny.

The fusion of tumor and myeloid cells was proposed at the beginning of the 20^th^ century by various German pathologists, such as Aichel, Dor, Hallion, and Kronthal, as cited with the first experimental results and discussion of spontaneous fusion *in-vivo* in 1968 [34]. This was based on the development of highly aggressive and metastatic tumors after grafting four different human cancers, with one of ovarian cancer origin (GW-127) showing hamster chromosomes, but also retention of human antigens [8], [9], [29], [30]. A series of subsequent studies described the transplantation of diverse human cancers to the cheek pouch of unconditioned (non-immunosuppressed) golden hamsters, and also showed metastases in their hamster hosts as early as 3–4 weeks after grafting, and the presence of both human and hamster markers within the cancer cells. The transplants displayed mostly hamster properties while retaining features of their human origin, including human chromosomes, isoenzyme patterns, antigens, and stathmokinetic properties in response to colchicine that was more compatible with human than hamster cells [30]–[33]. Over the course of about 15 years, while grafting more than 1200 primary human cancers to hamsters (cheek pouch site) or nude mice (subcutaneous site), 15 (1.25%) highly aggressive and metastatic tumors resulted from the hamster transplants [35]. These were derived from diverse solid and hematopoietic human tumors, and could be propagated *in-vitro* or *in-vivo* for years as permanent cell lines, showing rapid growth and metastatic features typical of a hamster tumor [10], [33], [35].

Since gene probes were not available then, it was only recently that FFPE tissues from these earlier transplants were subjected to FISH, PCR, and IHC methods to demonstrate the presence of both species' genetic markers and translation of human genes in some of these permanent transplants, even after years in the foreign, animal host [36], [37]. For example, the glioblastoma multiforme (GW-749) was reported in 1974 to be a human-hamster hybrid tumor based on retention of up to 15 human and many hamster chromosomes in the same malignant cells, as classified by Giemsa staining, even with definite identification of chromosomes karyotyped from the patient's lymphocytes, thus being a heterosynkaryon [33]. More recently, the GW-749 xenograft tumor was shown to have retained 7 transcribed human genes *(CD74, CXCR4, PLAGL2, GFAP, VIM, TP53, EGFR*), of which *CD74, CXCR4, and PLAGL2*, continued to be translated to their respective proteins that were visualized by IHC, as well as hamster X chromosome and human pancentromeric DNA in the same nuclei by FISH [36]. Surprisingly, these genes are known to have an association with malignancy and, in particular glial tumors, as well as *VIM* associated with mesenchymal cells. The transplants continued to express features of the original glioma tumor grafted, even after propagation in hamsters for ∼1 year [36].

Similar analyses were reported recently for two lymphomas grafted to hamsters [37], one of which was described in 1970 and shown to resemble its donor human tumor although gaining highly metastatic properties in the hamster [10]. FISH and PCR analyses showed that these two Hodgkin lymphoma-derived hybrid tumors displayed both hamster and human DNA in the same nuclei by FISH, while also retaining the human genes, *CD74, CXCR4, CD19, CD20, CD71, CD79b, and VIM*. It is noteworthy that the GB-749 glioblastoma hybrid tumor showed retention of glioma-related genes (*PLAGL2, GFAP*), whereas the lymphoma-derived hybrid tumor retained several B-cell antigen receptor (BCR)-related genes (*CD19, CD20, CD71, CD79b*). Three human genes, *CD74*, *CXCR4*, and *VIM*, were common to both the glioblastoma and lymphoma transplants. Both vimentin and CXCR4 are mesenchymal markers associated with epithelial-mesenchymal transition (EMT) whose genes were transcribed in all 3 hybrid tumors examined. It was also suggested that the heterosynkaryons of Hodgkin lymphoma with their Hodgkin Reed-Sternberg (HRS) cells retained their B-cell origin [37], confirming other evidence for a B-cell origin of this neoplasm [67], and again corroborated herein by gene probe analysis disclosing B-cell genes (*CD20, CD22*) in these specimens. As described, these tumors were observed within 2 weeks of their first transplantation, and showed evidence of metastasis in the hamster within 3–4 weeks [10], [37], suggesting that the hamster host's early response to the foreign tissue graft may have contributed to this process. Indeed, inflammation and wound healing are known to facilitate cell fusion [68]–[70].

In the current studies, we were interested in surveying the extent by which human DNA could be transferred and continuously transcribed in the hybrid tumors. Gene expression microarray analysis was performed using total RNA isolated from FFPE sections of these hybrid tumors, including two different transplant generations of GW-532. Unexpectedly, we detected a combined total of >3000 human genes amongst all of the samples, representing genes from all 23 pairs of human chromosomes, and found that 33 human genes were ubiquitously expressed in each of the 4 samples from the 3 tumors. Five of these genes encode transcription factors that are known to regulate cell growth and differentiation (*HOXB8, PPARA, POU2F2, ZFH2, ZNF580*), while another five encode cell adhesion and transmigration-associated proteins that are known to participate in tumorigenesis and/or metastatic invasion (*CDH3, FUT7, F11R, MUC3A*, and *SEMA3F*). Additional genes whose products can promote metastatic growth were also identified, including two signaling pathway enzymes (*PRKD2* and *ECEL1*), two apoptosis regulators (*CARD11* and *CFLAR*), the DNA repair and apoptosis regulator (*PARP15*), and the multidrug resistance gene (*ABCC6*). A representative publication for each of these 16 genes is provided in References S1. It is particularly noteworthy that published reports show that deregulated expression of either *PPARA* or *POU2F2* can promote oncogenic growth, the developmental function of *POU2F2* and *HOX* genes is to maintain cells in a less-differentiated state [71]–[80], and high expression of *ECEL1* gene was reported by Kawamoto et al [81] to associate with favorable prognosis in human neuroblastoma. A limitation of this evaluation, however, is the fidelity of the RNA extracted from these FFPE tissues, which were over 40 years old, emphasizing that only positive microarray results can be considered informative. This could explain why some of the genes identified in these specimens by PCR [36] were not identified by microarray analysis. In this study, however, both the DNA arrays and PCR identified the retention of transcribed human *F11R*, which codes for a junctional adhesion molecule. The other human gene detected by RT-PCR, α-satellite DNA, is present in the centromere of all human chromosomes, comprising the main structural component of heterochromatin. We should also note that the FFPE sections are of various transplant generations made over many years, and at various times studied *in vitro*. The populations are very uniform, not reflecting different cell populations morphologically. When the GB-749 glioma transplant was studied after transplantation, several generations showed the presence, in single cells, of both human and hamster chromosomes based on chromosome banding, and in fact compared to chromosomes identified in the donor patient's leukocytes. Since these were in single cells, we referred to these as heterosynkaryons. As such tumors were propagated for long periods, the cell population became very uniform, and there was never evidence of purely human tumor cells being propagated and maintained in serial passage.

Recently, the fusion of human bone marrow stromal cells with two human breast cancer cell lines indicated that the hybrid progeny were more metastatic than the parental breast cancers, and that analysis of coding single-nucleotide polymorphisms by RNA sequencing revealed genetic contributions from both parental partners, with between 1239 and 5345 genes from the parental cells retained in the fused cells [66]. However, these fused cells did not show long-term stability, but did retain breast cancer morphology [66]. In contrast, fusion of human cancer cells with normal stromal cells of murine mammary glands resulted in malignant tumors that had a sarcomatous appearance [82]. Two different human breast cancer cell populations injected into mice resulted in malignant cells that showed evidence of fusion in the mouse bone marrow, and were more extensively metastatic than the parental cell lines [83]. Similarly, two separate sets of genes that promote metastasis to bone and lung were combined via fusion of breast cancer cell lines, resulting in stable hybrids propagated long-term in cell culture and *in-vivo* [70]. Further, fusion of hematopoietic cells with human and murine epithelial ovarian cancer cells resulted in aggressive tumors of an epithelial phenotype retaining hematopoietic markers [84]. It is interesting that the chemokine receptor, CXCR4, which is a promigration marker, was expressed in the hybrid tumors, similar to our own experience of this chemokine's gene being transcribed in the three hybrid tumors studied here.

In our own experiments, the transcribed genes are known to be implicated in tumor progression to invasion and metastasis, including those involving EMT that is postulated to advance tumor cells to more malignant features [85], [86]. Recently, in fact, fusions of human lung cancer cells from cell lines and human bone marrow-derived mesenchymal stem cells, when co-cultured *in- vitro*, showed evidence of cell fusion and the convergence to a mesenchymal-like progeny with EMT and stem cell-like properties, even after injection into NOD/SCID mice [87]. Unfortunately, although considered by these authors as ‘spontaneous’ cell fusion, it is hardly spontaneous when 2 cell lines are grown together in culture, in contrast to growth of tumors that fuse *in-vivo* with unselected cells in their microenvironment. Nevertheless, such observations provide experimental evidence that in circumstances promoting horizontal gene transfer, whether or not truly spontaneous or the result of experimental conditions, new hybrid daughter tumor cells with new properties are generated, with features of more advanced malignancy [45], [46], [70], [82], [83]. Other experiments also have indicated that the progeny hybrid cells after fusion can acquire different properties than the parental cells [38], [43], [70], [82], [83], [88]. Thus, such fusion experiments may help further define genes and gene families participating in the evolution, change, and progression of human cancers by methods that are difficult to apply to humans or human tumor specimens directly.

It is intriguing that so many human genes, representing all individual human chromosomes, were transduced, transcribed, and retained permanently in our human-hamster hybrid tumors propagated *in- vivo* and *in- vitro*. Despite only 15 human chromosomes being identified by chromosome banding in various cells of two (5^th^ and 15^th^) transplant generations, which had the full complement of hamster as well as new marker chromosomes, of the GB-749 hybrid tumor derived from the human glioblastoma multiforme [36], the DNA array results indicate that a total of more than 3000 human genes were detected in a fourth generation passage in hamsters. This discrepancy provokes the speculation that human chromosomal fragments or genes could have translocated to hamster chromosomes, not unlike the DNA sequences (transposable, or “controlling elements”) described by McClintock in maize to relocate to other chromosomes in the genome [89], and known to regulate the expression of nearby genes. Over the ensuing 60 years, transposable elements, incorrectly referred to previously as ‘junk DNA”, have been confirmed to function in many animal species, including humans [90], even the insertion of a transposable element in the human genome that causes hemophilia A [91]. Retrotransposons (RNA transposons), or McClintock's “jumping genes”, may explain the retention of more human genes in these hybrid tumors than can be accounted for by the 15 human chromosomes identified by chromosome banding, and raises the question of whether similar events result generally with cell-cell fusions between tumor and normal stromal cells. These transposable elements are now understood to alter gene expression and promote genome evolution [92]. Indeed, lateral gene transfer can occur between microbes and animals [93], while retrotransposons jumping through the human genome can contribute to oncogenesis [90].

In order to reproduce this heterospecific hybridization experimentally, a murine melanoma was fused with hamster cheek pouch fibroblasts *in- vitro*, and the chromosomes of the daughter cells and their behavior *in- vivo* in hamsters and genetically-compatible mice were studied [94]. It was found that the murine-hamster hybrid tumor cells (confirmed karyologically) were more malignant in the hamster than the original murine melanoma was in mice, and that the hybrid tumor cells could not be propagated in genetically-compatible mice. Since the original murine melanoma could not grow in adult golden hamsters, the hamster genome came to dominate the genome of the hybrid tumor derived from the murine melanoma, retaining malignancy and metastasizability in hamsters but not in mice, while also losing expression of the melanin present in the original murine melanoma [94]. Evidently, the genetic contribution of the normal (fibroblast) cells governed the biological behavior and genetic features of the hybrid progeny, with the exception of malignancy and metastasizability derived from the murine melanoma. Similar experimental results of melanoma fusions with macrophages in mice have corroborated these findings, but where melanin was retained in the hybrid cells [46], [95], [96]. Thus, these various studies provide evidence of tumor progression after human-hamster, human-murine, and hamster-murine cell fusions.

The interpretation and relevance of these findings to human cancer are both challenging and stimulating. Does synkaryon formation and the progression of tumors to metastasizability constitute an isolated biological phenomenon without clinical relevance? Tumor heterogeneity has been a focus of interpretation and discussion since the beginnings of cancer histopathology, when diverse cell types and multinucleated giant cells were identified in the tumor and in its microenvironment. These gross cellular observations were then confirmed by genetic studies indicating a heterogeneity between different cells of the same tumor and between different metastases compared among themselves or to the primary tumor cells [63]–[65].

Cell-cell fusion may in fact be one mechanism of a more general process of intercellular DNA transfer. Supernatant from human tumor cell cultures or even cell-free DNA from human tumors or sera from cancer patients have been shown to induce tumors in recipient mice [27], [97], [98]. Other studies have suggested lateral transfer of non-cellular gene, RNA, or DNA via membrane-derived vesicles, exosomes, or other shed cell constituents [99], [100]. However, many of these experiments demonstrating oncogenicity utilized immortalized embryonic murine fibroblasts (NIH-3T3), which are known to be susceptible to transformation [98]. Nevertheless, human mutated gene sequences (e.g., *KRAS*) associated with the primary human cancers were transferred to the transformed murine fibroblasts by plasma DNA taken from human cancer patients, which then proved to be malignant in genetically-compatible mice [97]. Others have reported that circulating breast cancer cells exhibit epithelial and mesenchymal traits, with the latter indicating a more aggressive cell population [101]. The basis of this EMT, which has been discussed in many other models of malignancy [83], [85], [86], was not elucidated, but does stimulate questioning whether this could be due to DNA transfer, possibly via carcinoma-mesenchymal cell fusion, as already discussed in lung cancer x mesenchymal stem-cell fusion studies [101]. Indeed, it has been hypothesized that circulating cancer cells in humans express myeloid markers as a result of cell fusion [102].

These studies suggest that gene or DNA transfer between cells, forming recombinant gene hybrids, may not require cell-cell fusion and synkaryon formation. In fact, most of the recent studies implicating cell fusion are based on evidence of genetic markers of 2 different parental cells in the putative hybrid cell, in the absence of careful chromosome analyses showing a mixed karyotype in single nuclei. Hence, such experiments do not exclude gene transfer without actual synkaryon formation.

In conclusion, if cell-cell fusion is a basic biological process among many species and certain functions in humans [40], [45], [103], it is not unreasonable to expect that it would play an important role in oncogenesis [40]–[47], [88], accounting for genetic diversity within a single neoplasm or even between different tumors of the same patient. This would amend the long-held view of the clonal derivation of cancer cell populations [104], now emphasizing that horizontal gene interactions and cell-cell transfer also influence the development and change in cancer cell populations. But the major challenge is to prove that this mechanism is operative in cancer patients, for which evidence is accumulating in unique settings, such as in bone marrow transplantation transferring human chromosomes and genes to the recipients' tumors [105], and fusion of myeloma cells and osteoclasts in bone destruction [39], [106]. With the increasing interest in the crosstalk and exchange between cancer and stromal cells, including macrophages and leukocytes [43], [46], [69], the potential contribution of cell-cell fusion in the horizontal transfer of malignancy and other genes within a tumor deserves continued attention, and implies that this may be a basic biological process occurring between many different cell types both physiologically and in disease. In fact, there is evidence that novel transcriptomes can develop in hybrids that were not present in the parental cells [43], [70], [107]–[109].

Since the first evidence suggesting that cell fusion is a mechanism by which cancer cells become more diverse and progress to the advanced state of metastasis [33], [34], numerous experiments involving fusions of tumor x tumor, tumor x normal, and tumor x specific myeloid cells, as cited above and in recent reviews [40], [44]–[47], have made similar conclusions. However, it should be recognized that although revealing important attributes of cell-cell fusion in the recognition and plasticity of gene interactions and the development of hybrid daughter cells with phenotypic diversity, virtually all of these studies have utilized established cancer cell lines mixed either *in- vitro* or combined *in- vivo*, with the inherent limitations of cell line selection that may not be representative of the heterogeneous populations of primary tumors. This is emphasized by a publication that appeared while this article was under revision. It was reported that human pontine tumors obtained at autopsy and grafted orthotopically to immune-deficient mice either directly or via intermediate cell culture were different. Direct transplantation resulted in lethal tumors with murine characteristics, whereby the human tumor cells propagated first *in- vitro* remained human. Interesting, both populations retained the immunophenotype similar to human pontine glioma [110].

Finally, upon considering the literature on horizontal gene transfer, a distinction should be made between cell-cell fusion, resulting in nuclear merging of two genomes into a single cell, and the horizontal transfer of extracellular DNA as a basis of transduction. Two sets of gene markers derived from different parental cells in the nuclei of progeny cells do not, by themselves, prove one mechanism or the other. These processes should be distinguished in order to devise potential therapeutic strategies to control the horizontal transfer of DNA between malignant and stromal cells in their microenvironment, or to adapt the process to enhance anticancer immunity.

## Materials and Methods

### Tumor xenografts

***GW-532*** [10]: A male's left axillary Hodgkin lymphoma containing Hodgkin Reed Sternberg (HRS) cells was grafted to the cheek pouches of adult, unconditioned golden hamsters (*Mesocricetus auratus*), and the resulting tumor was serially passaged in hamsters for >6 years [37]. The transplants were morphologically similar to portions of the original donor specimen, even with HRS cells being identified as early as 17 days after the initial transplantation. This and all subsequent transplant generations showed widespread metastases from the cheek pouch grafts. Transplant generations 2 and 34 were used for genetic analyses. ***GW-584*** [37]: This was a transplant line established in hamster cheek pouches from the mediastinal Hodgkin lymphoma of a male, also showing HRS cells, and propagated for >5 years. The serial transplants were similar morphologically to the first generation xenograft. The first evidence of metastasis to all major organs and lymph nodes was observed as early as 21 days from the initial grafting, and continued in all subsequent transplant generations, regardless of transplant site. Transplant generation 28 was used for the current studies. ***GB-749*** [36]: As described earlier [33], this glioblastoma multiforme specimen from an adult female was successfully grafted to the cheek pouch of 1 of 9 unconditioned, adult golden hamsters; this tumor appeared in 14 days and killed the recipient due to widespread metastasis by 4 weeks. This aggressive and rapid growth was continued upon serial passage to other hamsters, showing metastases to all major organs regardless of transplant site in the hamster. Morphologically, the transplant was more uniform and anaplastic than the patient's tumor, but showed the pseudopalisading, lobulated pattern and/or sheets of cells similar to the original patient tumor, even after serial transplantation for >2 years [33], [36]. In the original description of this tumor line, karyological studies showed that the malignant cells were heterosynkaryons composed of both human and hamster chromosomes, including 15 human chromosomes (numbers 1, 2, 3, 5, 6, 7, 9, 10, 11,12,13,15, 16,18, and 21, with 6 being identical to the lymphocyte chromosomes of the donor patient) [36]. This was the first experimental evidence of spontaneous *in-vivo* fusion of human tumor and an animal host's normal cells [103], as corroborated by heterosynkaryon formation in the daughter cells.

Recent studies of the GW-532, GW-584, and GB-749 transplants by FISH, RT-PCR, and IHC showed that at least 7 human genes were transcribed in each of these tumor lines, with 3 genes being translated to produce their proteins in the GB-749 line [36]. FISH experiments confirmed the presence of both human and hamster DNA in the same malignant cells in all 3 transplant lines [36], [37].

All FFPE tissues were more than 40 years old, and stored at room temperature.

### RNA samples and isolation

FFPE tissues of selected samples (Table 1) were sliced into 4- to 5-µm sections. For each sample, four sections were combined for one total RNA preparation using Qiagen RNeasy FFPE Kit (Qiagen, Germantown, MD) according to the manufacturer's instructions. Briefly, the sections were deparaffinized, followed by incubation with proteinase K at 56°C for 15 min. After inactivation of the proteinase K, the mixture was centrifuged, from which the supernatant was treated with DNase I at room temperature for 15 min, then transferred to a column supplied in the kit. After several washes, the RNA was eluted with 22 µL of RNase-free water. The same procedure was used for preparing total RNA from 4×10^6^ cells of CCL-49, a Syrian golden hamster melanoma cell line purchased from ATCC and cultured in McCoy's 5A medium supplemented with Na-Pyruvate, Glutamax, Penstrep, and 10% FBS.

### RNA quality control

Immediately prior to cDNA synthesis, the purity and concentration of RNA samples were determined from OD_260/280_ readings using a dual beam UV spectrophotometer, and RNA integrity was determined by capillary electrophoresis using the RNA 6000 Nano Lab-on-a-Chip kit and the Bioanalyzer 2100 (Agilent Technologies, Santa Clara, CA), as per the manufacturers' instructions.

### cDNA synthesis and labeling

RNA (50 ng each sample) was converted to cDNA, amplified by the Single Primer Isothermal Amplifcation (SPIA) method, fragmented and labeled with biotin using Ovation Pico WTA System v2 and Encore Biotin Module kits according to the manufacturer's instructions (NuGEN, San Carlos CA).

### Oligonucleotide array hybridization and analysis

Fragmented, biotinylated cDNA was hybridized for 20 h at 45°C to GeneChip Human U133_X3P Arrays (Affymetrix, Santa Clara CA). The Human U133_X3P arrays contain over 61,000 oligonucleotide probe sets that are specifically designed to interrogate 3′ regions in more than 47,000 different gene transcripts. Arrays were washed and stained with phycoerythrein-conjugated streptavidin (Life Technologies, Carlsbad, CA) in a Fluidics Station 450 (Affymetrix), according to the manufacturer's recommended procedures. Fluorescence intensities were determined using a GCS 3000 7G high-resolution confocal laser scanner, and analyzed using the programs in AGCC and Expression Console (Affymetrix). MAS 5.0 and RMA Quality Control outputs from Expression Console were used to monitor sample and array performance and identify potential outlier arrays; outlier evaluation was also performed by Principal Components Analysis in GeneMaths XT (Applied Maths, Austin TX).

### Data analysis

Signal expression values and detection *P*-values were generated by MAS 5.0 [111]–[113], after which unannotated probe sets, as well as probe sets with no signal value greater than the median signal for AFFX spike-in controls with all Absent Detection Calls, were omitted from further analysis. Because an intact hamster cell line (CCL-49) control RNA sample was included for comparison with the four human-hamster hybrid FFPE samples, all remaining signal values for the hamster cell line sample were multiplied by the ratio of the median signal in all FFPE hybrid samples for AFFX spike-in control probe sets called present in all samples divided by the median signal for the same probe sets in the hamster CCL-49 sample. Human transcripts were considered positive in human-hamster hybrid FFPE samples if (*i*) a probe set signal exhibited a 2-fold or greater increase in any FFPE hybrid sample compared to the CCL-49 sample, (*ii*) the fold change was greater than 2 standard deviations for that probe set across the FFPE samples, and (*iii*) was called present (P) or marginal (M) for at least one or two FFPE samples (as indicated in the text).

Unsupervised hierarchical clustering and heat map generation were performed in GeneMaths XT (Applied Maths, Belgium) following row mean centering of log2 transformed MAS 5.0 signal values; probe set and sample clustering were performed by Complete Linkage based on Euclidean distance.

Gene annotation and gene ontology information were obtained from the National Center for Biotechnology Information (www.ncbi.nlm.nih.gov), NetAffx (www.affymetrix.com), and the the Gene Ontology Consortium (http://amigo.geneontology.org). Pathway annotation and enrichment analysis were performed on-line using WebGestalt (http://bioinfo.vanderbilt.edu/webgestalt). Significant enrichment of specific GO categories or KEGG pathways in each comparison was estimated by hypergeometric tests or *chi* square tests. Additional bioinformatics analysis was performed using DAVID [56], [57] and PharmGKB [114].

The data files have been deposited in the Gene Expression Ombibus, and can be viewed at http://www.ncbi.nlm.nih.gov/geo/query/acc.cgi?acc=GSE58277.

### PCR and one-step reverse transcription PCR

Genomic DNA was isolated from FFPE tissues using QIAamp DNA FFPE Tissue Kit (Qiagen, Germantown, MD) and from Raji or hamster CCL-49 cells using DNeasy Tissue Kit (Qiagen), according to the manufacturer's instructions. Total RNA was isolated from FFPE tissues using FFPE RNA/DNA Purification Plus Kit (Norgen Biotek, Thorold, Ontario, Canada) and from human HepG2 or hamster CCL-49 cells using TRIzol Reagent (Life Technologies, Grand Island, NY).

PCR was performed using a pair of primers (forward: 5' CAT CAC AAA GAA GTT TCT GAG AAT GCT TC 3'; reverse: 5' TGC ATT CAA CTC ACA GAG TTG AAC CTT CC 3') directed to a conserved region of the 17l-bp monomer of human α-satellite DNA [48] under the following conditions: 94°C/30 sec, 60°C/30 sec, 72°C/30 sec for 45 or 50 cycles. Genomic DNA from human Raji or hamster CCL-49 cells served as positive and negative controls, respectively.

One-step reverse transcription PCR was performed to assess the presence of mRNA transcripts of the *F11R* gene using SuperScript III One-Step RT-PCR System (Life Technologies) under the following conditions: one cycle of cDNA synthesis (55°C/30 min) and 50 cycles of PCR (94°C/15 sec, 56°C/30 sec, 68°C/30 sec). The pair of primers (UniSTS database) used were: forward: 5′ ACT GGG GTC CTT CCA TCT CT 3′; reverse: 5′ CAC AAC AAG AGC TCC CAT T 3′. Total RNA from human HepG2, which is known to express *F11R* [49], and hamster CCL-49 cells served as positive and negative controls, respectively.

## Supporting Information

Figure S1 **Additional one-step reverse transcription PCR.** The mRNA transcripts of *the F11R* gene were detectable in GW-532 generation 11 (lane 1), GW-584 generation 3 (lane 2), and the positive control of HepG2 cells (lane 5), whereas the target 141-bp was apparently absent in the negative control of hamster melanoma CCL-49 cells (lane 4). The experimental conditions and the nominal amount of RNA used for each sample are indicated.
(PPTX)

Table S1 **All probe sets found to be expressed in any of the four human-hamster hybrid samples.**
(XLSX)

Table S2 **All probe sets found to be expressed in all four human-hamster hybrid samples.**
(XLSX)

Table S3 **Notable transcripts of genes present in all four hybrid samples.**
(DOCX)

Table S4 **All probe sets found to be expressed in all four human-hamster hybrid samples using a relaxed detection** ***p*** **-value.**
(XLSX)

Table S5 **Functionally related pathways common to the four human-hamster hybrid samples.**
(XLSX)

Table S6 **DAVID functional annotation clusters in the Table S1 gene set that are common to the four human-hamster hybrid samples.**
(XLSX)

References S1 **A representative publication of each gene or its expressed protein as listed in Table S3 is provided.**
(DOCX)
