# Supplementary material for: In-Vivo Fusion of Human Cancer and Hamster Stromal Cells Permanently Transduces and Transcribes Human DNA
Source: PLoS One. 2014 Sep 26;9(9):e107927. doi: 10.1371/journal.pone.0107927 (PMC4178054; doi:10.1371/journal.pone.0107927)
Supplement: Figure S1 — Additional one-step reverse transcription PCR. The mRNA transcripts of the F11R gene were detectable in GW-532 generation 11 (lane 1), GW-584 generation 3 (lane 2), and the positive control of HepG2 cells (lane 5), whereas the target 141-bp was apparently absent in the negative control of hamster melanoma CCL-49 cells (lane 4). The experimental conditions and the nominal amount of RNA used for each sample are indicated. (PPTX) [file pone.0107927.s001.pptx]

## Slide 1
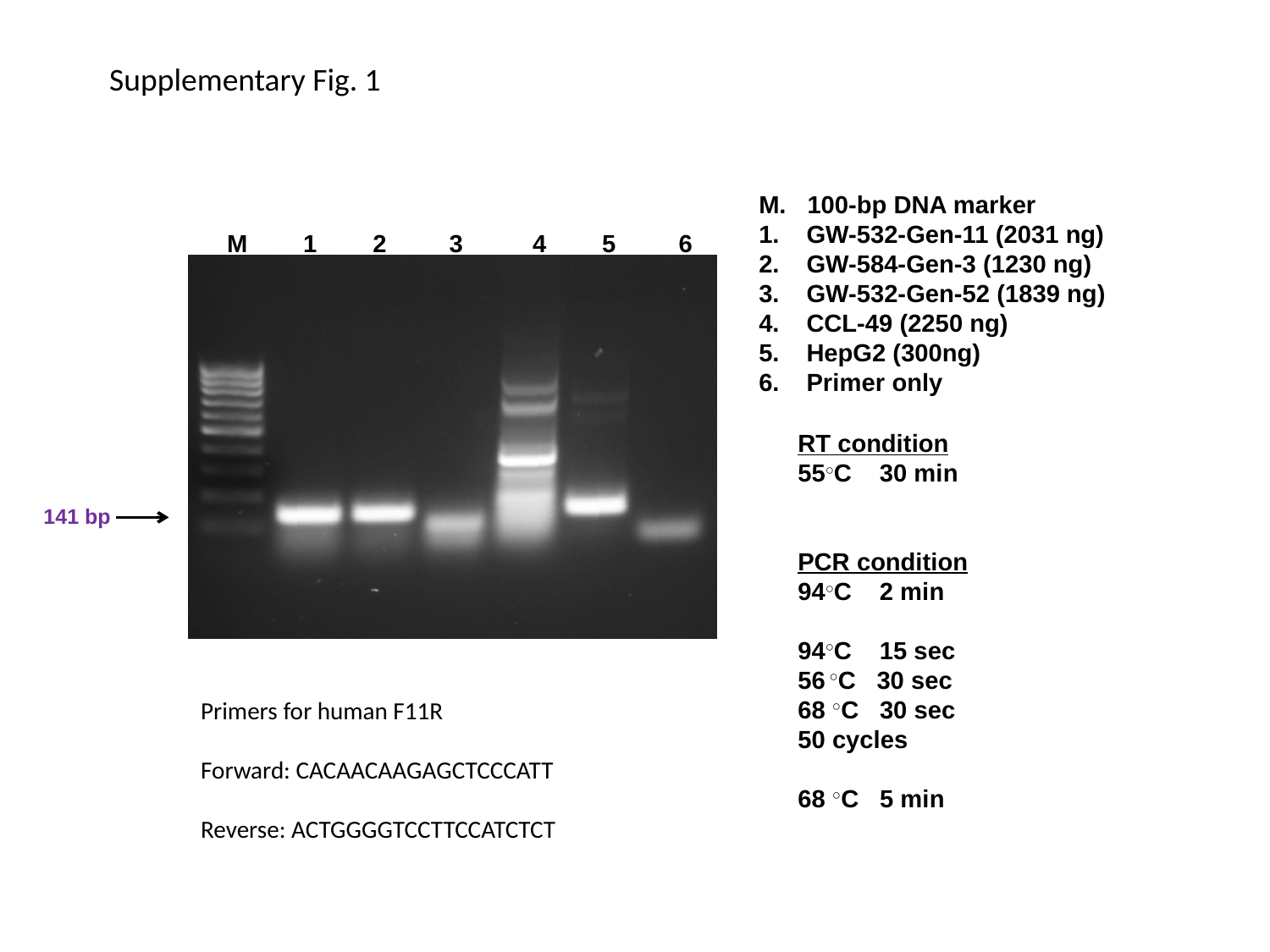

Supplementary Fig. 1
M. 100-bp DNA marker
GW-532-Gen-11 (2031 ng)
GW-584-Gen-3 (1230 ng)
GW-532-Gen-52 (1839 ng)
CCL-49 (2250 ng)
HepG2 (300ng)
Primer only
 M 1 2 3 4 5 6
RT condition
55○C 30 min
PCR condition
94○C 2 min
94○C 15 sec
56 ○C 30 sec
68 ○C 30 sec
50 cycles
68 ○C 5 min
141 bp
Primers for human F11R
Forward: CACAACAAGAGCTCCCATT
Reverse: ACTGGGGTCCTTCCATCTCT
