# Supplementary material for: In-Vivo Fusion of Human Cancer and Hamster Stromal Cells Permanently Transduces and Transcribes Human DNA
Source: PLoS One. 2014 Sep 26;9(9):e107927. doi: 10.1371/journal.pone.0107927 (PMC4178054; doi:10.1371/journal.pone.0107927)
Supplement: Table S3 — Notable transcripts of genes present in all four hybrid samples. (DOCX) [file pone.0107927.s004.docx]

Table S3. Notable transcripts of genes present in all four hybrid samples.

| Gene | Protein | Function | Reference^a^ |
| --- | --- | --- | --- |
| *HOXB8* | Homeobox B8 | Transcriptional factor | S1 |
| *POU2F2* | POU class 2 homeobox 2; Oct-2^b^ | Transcriptional factor | S2 |
| *ZFHX2* | zinc finger homeodomain-2 | Transcriptional factor | S3 |
| *PPARA* | peroxisome proliferator-activated receptor alpha | Transcriptional factor | S4 |
| *ZNF580* | Zinc finger protein 580 | Transcriptional factor | S5 |
| *CDH3* | P-cadherin | Tumor progression | S6 |
| *FUT7* | fucosyltransferase 7 | Metastasis | S7 |
| *F11R* | Junctional adhesion molecule (JAM)-A^b^; JAM-1^b^ | Tumor proliferation | S8 |
| *MUC3A* | Mucin 3A | Cell-migration stimulator | S9 |
| *SEMA3F* | semaphorin 3F | Tumor-suppressor | S10 |
| *PRKD2* | Protein kinase D2 | Metastasis | S11 |
| *ECEL1* | Endothelin-converting enzyme-like 1 | Zinc metallopeptidase | S12 |
| *CARD11* | caspase recruitment domain family, member 11 | Oncogene | S13 |
| *CFLAR* | c-FLIP | Apoptosis regulator | S14 |
| *PARP15* | poly (ADP-ribose) polymerase family, member 15; BAL3^b^ | Tumor promoting factor | S15 |
| *MRP6* | multidrug resistance associated protein 6; ABCC6^b^ | Multidrug resistance | S16 |

^a^A representative publication of each gene or its expressed protein is provided in the Reference S1 file.

^b^Alternative designation.
