# Supplementary material for: In-Vivo Fusion of Human Cancer and Hamster Stromal Cells Permanently Transduces and Transcribes Human DNA
Source: PLoS One. 2014 Sep 26;9(9):e107927. doi: 10.1371/journal.pone.0107927 (PMC4178054; doi:10.1371/journal.pone.0107927)
Supplement: References S1 — A representative publication of each gene or its expressed protein as listed in Table S3 is provided. (DOCX) [file pone.0107927.s008.docx]

**Supporting References**

S1. Greer JM, Capecchi MR (2002) *HOXB8* is required for normal grooming behavior in mice. Neuron 33: 23-34.

S2. Latchman DS (1996) The Oct-2 transcription factor. Int J Biochem Cell Biol 28: 1081-1083.

S3. Komine Y, Takao K, Miyakawa T, Yamamori T (2012) Behavioral abnormalities observed in Zfhx2-deficient mice. PLoS One 7: e53114.

S4. Van Raalte DH, Li M, Pritchard PH, Wasan KM (2004) Peroxisome proliferator-activated receptor (PPAR)-alpha: A pharmacological target with a promising future. Pharm Res 21: 1531-1538.

S5. Luo Y1, Hu W, Xu R, Hou B, Zhang L, et al. (2011) ZNF580, a novel C2H2 zinc-finger transcription factor, interacts with the TGF-β signal molecule Smad2. Cell Biol Int 35:1153-1157.

S6. Paredes J, Figueiredo J, Albergaria A, Oliveira P, Carvalho J, et al. (2012) Epithelial E- and P-cadherins: role and clinical significance in cancer. Biochim Biophys Acta. 1826: 297-311.

S7. Läubli H, Stevenson JL, Varki A, Varki NM, Borsig L (2006) L-selectin facilitation of metastasis involves temporal induction of Fut7-dependent ligands at sites of tumor cell arrest. Cancer Res 66:1536-1542.

S8. Goetsch L, Haeuw JF, Beau-Larvor C, Gonzalez A, Zanna L, et al. (2013) A novel role for junctional adhesion molecule-A in tumor proliferation: modulation by an anti-JAM-A monoclonal antibody. Int J Cancer. 132:1463-1474.

S9. Ho SB, Dvorak LA, Moor RE, Jacobson AC, Frey MR, et al. (2006) Cysteine-rich domains of muc3 intestinal mucin promote cell migration, inhibit apoptosis, and accelerate wound healing. Gastroenterology 131:1501-1517.

S10. Kusy S, Nasarre P, Chan D, Potiron V, Meyronet D, et al. (2005) Selective suppression of in vivo tumorigenicity by semaphorin SEMA3F in lung cancer cells. Neoplasia 7: 457-465.

S11. Bernhart E, Damm S, Wintersperger A, DeVaney T, Zimmer A, et al. (2013) Protein kinase D2 regulates migration and invasion of U87MG glioblastoma cells in vitro. Exp Cell Res 319: 2037-2048.

S12. Valdenaire O, Rohrbacher E, Langeveld A, Schweizer A, Meijers C (2000) Organization and chromosomal localization of the human ECEL1 (XCE) gene encoding a zinc metallopeptidase involved in the nervous control of respiration. Biochem J. 346 Pt 3: 611-616.

S13. Lenz G, Davis RE, Ngo VN, Lam L, George TC, et al. (2008) Oncogenic CARD11 mutations in human diffuse large B cell lymphoma. Science 319: 1676-1679.

S14. Safa AR (2012) c-FLIP, a master anti-apoptotic regulator. Exp Oncol 34:176-184.

S15. Welsby I, Hutin D, Leo O (2012) Complex roles of members of the ADP-ribosyl transferase super family in immune defences: looking beyond PARP1. Biochem Pharmacol 84: 11-20.

S16. Belinsky MG, Chen ZS, Shchaveleva I, Zeng H, Kruh GD (2002) Characterization of the drug resistance and transport properties of multidrug resistance protein 6 (MRP6, ABCC6). Cancer Res 62: 6172-6177.
